# Distinguishing between straight and curved sounds: Auditory shape in pitch, loudness, and tempo gestures

**DOI:** 10.3758/s13414-023-02764-8

**Published:** 2023-09-18

**Authors:** Sven-Amin Lembke

**Affiliations:** Cambridge School of Creative Industries, Anglia Ruskin University, Cambridge, UK

**Keywords:** Sound gesture, Crossmodal correspondence, Audiovisual, Pitch, Loudness, Tempo

## Abstract

Sound-based trajectories or sound gestures draw links to spatiokinetic processes. For instance, a gliding, decreasing pitch conveys an analogous downward motion or fall. Whereas the gesture’s pitch orientation and range convey its meaning and magnitude, respectively, the way in which pitch changes over time can be conceived of as gesture shape, which to date has rarely been studied in isolation. This article reports on an experiment that studied the perception of shape in uni-directional pitch, loudness, and tempo gestures, each assessed for four physical scalings. Gestures could increase or decrease over time and comprised different frequency and sound level ranges, durations, and different scaling contexts. Using a crossmodal-matching task, participants could reliably distinguish between pitch and loudness gestures and relate them to analogous visual line segments. Scalings based on equivalent-rectangular bandwidth (ERB) rate for pitch and raw signal amplitude for loudness were matched closest to a straight line, whereas other scalings led to perceptions of exponential or logarithmic curvatures. The investigated tempo gestures, by contrast, did not yield reliable differences. The reliable, robust perception of gesture shape for pitch and loudness has implications on various sound-design applications, especially those cases that rely on crossmodal mappings, e.g., visual analysis or control interfaces like audio waveforms or spectrograms. Given its perceptual relevance, auditory shape appears to be an integral part of sound gestures, while illustrating how crossmodal correspondences can underpin auditory perception.

## Public Significance Statement

We often hear sounds that convey spatial or motional relationships, such as when a sound descending in pitch seems to imply a downward motion or fall. This study demonstrates how listeners reliably perceive and express sounds with ascending or descending pitch or increasing or decreasing loudness as analogous visual shapes, e.g., straight vs. curved lines, whereas it could not be reliably achieved for accelerating or decelerating tempo. Such differences in sonic shape help us better understand how we extract meaning from changes in speech intonation or sound signalization in everyday environments; they can inform the design of related applications and inspire creative uses in sound design for audiovisual media like films and also musical composition.

## Introduction

Picture this scene from a Looney Tunes animation: in the endless chase between the Road Runner and him, Wile E. Coyote races toward the edge of a cliff at perilous speed. Even if viewers unfamiliar with this animation classic closed their eyes, they will *hear* the coyote fall off the cliff. This is because viewers hear a sound with continuously descending pitch, one of the tried-and-tested recipes sound designers and film composers use to convey a sense of downward motion. Although this audiovisual correspondence between descending pitch and vertical position is one of the most iconic and frequent, it represents only one out of many examples of *sound gestures*, in which a time-variant auditory property conveys an analogous spatiokinetic trajectory or process.

In psychological research, such audiovisual correspondences relate to the wider study of crossmodal correspondences or associations, which have received increasing attention in past decades (Deroy & Spence, 2016; Marks, 1996; Spence, 2011) and concern stimulus mappings across sensory modalities that are experienced as ‘natural’, e.g., low and high auditory pitches mapping to low and high vertical positions, respectively. Whereas such correspondences resemble metaphors that can be conceived of voluntarily by conscious association and thus involve the decisional level of processing, many reported crossmodal correspondences operate at even the pre-attentive, perceptual level and therefore lie beyond conscious control. To isolate the contribution of the perceptual from the decisional factors, experimental methods commonly control for response bias and mediating variables and employ speeded-classification tasks. Such tasks focus on a single sensory modality while the unattended modality is varied to be either crossmodally congruent or incongruent. Although these paradigms confirm crossmodal correspondences’ perceptual origin when the congruent mapping exhibits superior task performance, they do often not clarify how these correspondences arise, whether they apply in similar ways across wider stimulus ranges and what crossmodal functions underlie them (Anikin & Johansson, 2019; Marks, 1996; Parise, 2016). To “measure the shape of the mapping function” (Parise, 2016, p. 15), experiments must consider a wider sampling of stimuli, where direct adjustment tasks have been more efficient (e.g., Guzman-Martinez, Ortega, Grabowecky, Mossbridge, & Suzuki, 2012; Orchard Mills, Alais, & Van der Burg, 2013; Stevens & Marks, 1965).

In creative or communicational applications, gestures that are expressed through sound are expected to draw extrasonic links to spatial or kinetic actions, either concerning actual motions by performing musicians or implied metaphorical ones (see Jensenius, Wanderley, GodØy, & Leman, 2010). Only gestures in the metaphorical sense are considered here, which is not to say that some may in fact involve crossmodal correspondences at the perceptual level. The use of such sound gestures extends beyond sound design for film or television and assumes importance to music in general (e.g., Goodchild, Wild, & McAdams, 2019; Hatten, 2004). Hatten (2004, p. 95) defined such gestures as “significant energetic shaping of sound through time”, while for the genre of electroacoustic music, Smalley (1997, p. 113) established a similar “notion of gesture as a forming principle [that] is concerned with propelling time forwards, with moving away from one goal towards the next goal in the structure—the energy of motion expressed through spectral and morphological change”. Similar to how music has been argued to allude to motion more generally (Johnson & Larson, 2003), sound gestures contribute to the formation of sound shapes (Blackburn, 2011; Lembke, 2018; Smalley, 1986, 1997), which in turn can be used to develop musical form and discourse.

Employing Smalley’s notion of a goal-oriented process, a single sound gesture can be conceived as representing one informational unit, based on which the identity of a gesture emerges at a higher-level, *meso* timescale, whereas the temporal evolution defining its morphology takes place at a lower-level, *micro* scale (Godøy, 2006). With regard to what constitutes gesture identity, a wider body of research becomes relevant: this includes the typology of gestures in the context of spectromorphology (Smalley, 1997) or temporal semiotic units (Delalande, 1996; Frey, Daquet, Poitrenaud, Tijus, Fremiot, Formosa, & Prod’Homme..., 2009)^1^, with both frameworks describing the temporal evolution of sound features in terms of motion types, such as *ascent*, *fall*, *deceleration*, *rotation* or *flotation*. Musical gestures also relate to psychological theories of embodiment (Godøy, 2006; Jensenius, Wanderley, Godøy, & Leman, 2010).

Apart from gesture type or category, such as decreasing pitch symbolizing a *fall*, previous research attempted to identify and describe the crossmodal underpinnings. To measure the relationship from sound to gesture, various methods have been employed, ranging from descriptions of mental imagery (Eitan & Granot, 2006), free-hand drawings (Godøy, Haga, & Jensenius, 2006; Blackburn, 2013; Küssner & Leech-Wilkinson, 2014; Engeln & Groh, 2021; Athanasopoulos & Moran, 2013), associations to visual shapes (Merer, Aramaki, Ystad, & Kronland-Martinet, 2013; Thoret, Aramaki, Kronland-Martinet, Velay, & Ystad, 2014; Lembke, 2018) to motion-capture recordings (Caramiaux, Bevilacqua, & Schnell, 2010; Nymoen, Caramiaux, Kozak, & Torresen, 2011; Nymoen, Torresen, Godøy, & Jensenius, 2012; Küssner, Tidhar, Prior, & Leech-Wilkinson, 2014; Lemaitre, Scurto, Françoise, Bevilacqua, Houix, & Susini, 2017). Gesture identity has been shown to depend on the auditory parameter in question as well as the crossmodal orientation and magnitude.

Given its high prevalence in crossmodal correspondences (Deroy & Spence, 2016; Spence, 2011), pitch represents the most reliable auditory parameter to feature in sound gestures (Eitan & Granot, 2006; Küssner & Leech-Wilkinson, 2014; Küssner, Tidhar, Prior, & Leech-Wilkinson, 2014; Lemaitre, Jabbari, Misdariis, Houix, & Susini, 2016; Lemaitre, Scurto, Françoise, Bevilacqua, Houix, & Susini, 2017; Nymoen, Caramiaux, Kozak, & Torresen, 2011; Nymoen, Torresen, Godøy, & Jensenius, 2012), where its association with verticality or elevation is widespread, both in terms of the use of the verbal labels *low* and *high* and its ecological underpinnings (Parise, Knorre, & Ernst, 2014). As pitch (height) and timbral brightness vary along spectral frequency, both can evoke sound gestures in similar ways (Nymoen, Caramiaux, Kozak, & Torresen, 2011; Nymoen, Torresen, Godøy, & Jensenius, 2012), while in a related context, pitch and brightness contours can be considered equivalent when they share the same low-to-high mapping (McDermott et al., 2008).

Also continuous loudness variations, as in increasing or decreasing loudness ramps, give rise to sound gestures, although the spatiokinetic associations vary more widely, occupying horizontal or vertical space (Küssner & Leech-Wilkinson, 2014; Nymoen, Caramiaux, Kozak, & Torresen, 2011; Nymoen, Torresen, Godøy, & Jensenius, 2012), spatial depth (Eitan & Granot, 2006) or more kinetic measures like pressure (Küssner & Leech-Wilkinson, 2014), velocity, acceleration or energy (Caramiaux, Bevilacqua, & Schnell, 2010; Nymoen, Caramiaux, Kozak, & Torresen, 2011; Nymoen, Torresen, Godøy, & Jensenius, 2012).

Not an auditory parameter per se but no less relevant as a musical parameter, tempo variations, as in accelerations and decelerations, also bear potential to convey sound gestures. For one, the close association of tempo or rhythmic deceleration to a kinetic equivalence in velocity has been modeled (Friberg & Sundberg, 1999), while similar time models apply to both orientations of tempo variation in musical phrasing (Repp, 1992). Although in one case tempo has been considered in the context of human-arm gestures in response to sound (Küssner, Tidhar, Prior, & Leech-Wilkinson, 2014), it did not represent a gestural parameter itself but rather determined the time course along other auditory parameters, a notable distinction to the current study.

In summary, sound gestures can involve the parameters pitch, timbre, loudness, and possibly also tempo. Previous research identified the underlying crossmodal mappings (e.g., verticality, velocity), their orientation (e.g., up vs. down, deceleration vs. acceleration), and assessed their reliability. Returning to the example of Wile E. Coyote and what constitutes the identity of a gesture, the downward orientation along pitch classifies the gesture’s *type* or *category* as a ‘fall’, while the extent of the pitch variation (e.g., 2 octaves) and/or its duration represents the gesture’s *magnitude*. Importantly, these properties only delimit gesture identity without conveying how the fall occurs over time, a property that here will be introduced as the gesture’s *shape*.

A pitch gesture that decreases at about the same rate throughout its duration assumes one shape, e.g., resembling a straight line, whereas pitch decreasing gradually at first and ending steeply forms another shape, e.g., a logarithmic curve. Such monotonic decreases from point of departure to arrival can trace a variety of paths and shapes. Although differences in the time course for otherwise equivalent pitch gestures has been shown to indeed affect human gestural expression (Küssner, Tidhar, Prior, & Leech-Wilkinson, 2014), the isolated role of how gestures are shaped over time has not been studied, notably, also for more basic, uni-directional and uni-parametric gestures (e.g., a monotonic upward trajectory along pitch). This study sought to fill this knowledge gap by focusing on shape-related aspects in uni-directional gestures along either pitch, loudness, or tempo.

Gesture shape arises from the physical scaling of sound properties that underpins crossmodal association. For instance, the laws of mechanics state that a mass falls at constant acceleration based on gravitational force, which in turn corresponds to either a quadratic decrease in elevation or a linear increase in velocity over time. Whereas both represent accurate descriptions of the physical motion involved, using one or the other as input to a crossmodal mapping would without doubt lead to different sonic outcomes. The current study explored such differences along the perceptual parameters pitch, loudness, and tempo based on the consideration of physical scales employed in common applications. The description of sound-gesture shape employed analogous visual trajectories, comparable to the aforementioned methods to match motion-related sounds to static or dynamic visual shapes or, more generally, compare sound gestures to drawings or recorded motion trajectories.

The auditory gestures studied stem from common sound-based applications. For instance, time-based visual representations of frequency, as in spectrograms or Western musical notation, concern mappings of low to high frequencies from bottom to top vertically, while the horizontal dimension signifies time. Of importance to the design of such representations, if a visual interface like a spectrogram depicted a straight line increasing in frequency over time, what shape would the auditory pitch gesture correspond to? This would depend on the frequency scaling used, where equivalent rectangular bandwidth (ERB) rate (Moore & Glasberg, 1983) could be hypothesized to render the perceptually most ‘linear’ trajectory, as this psychoacoustically derived scale approximates the frequency resolution of human hearing. Likewise, different shapes for loudness gestures result from different scalings of signal amplitude, where in audio-editing software, for instance, linear or curved shapes are commonly applied as fade-in/out control functions. Do the shapes of these visual control functions, when applied to raw amplitude as opposed to its logarithmic transformation into level in decibels, match the auditory shape in terms of loudness variation? Thirdly, in automobiles, parking aids are commonly sonified, in that the time rate or tempo of repeated beeps may increase as the distance to an obstacle decreases. If sound designers aimed to convey most intuitively by how much drivers need to decelerate to avoid collision, they would have to determine the best crossmodal mapping between motion deceleration and tempo acceleration.

**Table 1:** Parameter ranges across frequency, sound pressure level (SPL), and bursts per minute (BPM) used in the pitch, loudness, and tempo gestures, respectively

Parameter	Parameter ranges				
	Bottom		Anchor		Top
Pitch gesture		lower		higher	
Frequency (Hz)	250	$\leftarrow$ $\rightarrow$	**1,000**	$\leftarrow$ $\rightarrow$	4,000
Loudness gesture		*softer*		*louder*	
SPL (dB)	46	$\leftarrow$ $\rightarrow$	**64**	$\leftarrow$ $\rightarrow$	82
Tempo gesture		*slower*		*faster*	
BPM	180	$\leftarrow$ $\rightarrow$	360	$\leftarrow$ $\rightarrow$	720
Median duration (s)		**3**.**51**		**1**.**80**	

Gestures along each parameter were investigated at two levels, e.g., a *lower* and a *higher* pitch range, spanning bottom-to-anchor and anchor-to-top ranges, respectively. Parameter values in *bold* also represent the base settings for gestures along the remaining parameters

In conclusion, the auditory shape of sound gestures can be argued to relate to various application scenarios and will be studied here in greater breadth. For a given auditory parameter, it is hypothesized that listeners can distinguish and describe differences in gesture shape that arise from differences in the physical scales that underlie them. Although previous psychophysical scales may represent probable candidates to yield ‘linearly’ scaled perceptions, the aim is to also establish how other scalings may resemble non-linear, curved shapes. The next section presents the method to investigate how differences in gesture shape for pitch, loudness, and tempo gestures are perceived for the scenarios discussed above in a perceptual experiment. This is followed by a section that reports the experiment’s results, which are thereupon discussed and contextualized in the final two sections.

## Method

A perceptual listening experiment investigated differences in perceived shape for uni-directional sound gestures along the auditory parameters pitch, loudness or tempo. In each case, four underlying physical scales were considered (e.g., linear, logarithmic, exponential). A crossmodal matching task was used to express and measure differences along auditory shape as analogous differences along visual shape.

In order to identify potential contextual bias among the four scale variants, each was evaluated across different stimulus contexts. For a more representative study of the perception of gesture shape, gestures furthermore involved increasing and decreasing orientations and also spanned different ranges across frequency, sound level, and tempo or duration.

Given the previously established notion of metaphorical sound gestures (Hatten, 2004; Jensenius, Wanderley, Godøy, & Leman, 2010; Smalley, 1997), the method employed here sought to identify and describe crossmodal mappings in a free, associative context. Whether the observed mappings were in fact founded on crossmodal correspondences at the perceptual level (Deroy & Spence, 2016; Spence, 2011) remained outside the focus of the current study.

### Stimuli

All stimuli were pre-produced in digital PCM format at 44.1 kHz sampling rate and 24-bit dynamic resolution. The gesture signals were composed of band-pass filtered pink noise using a second-order Butterworth design. A relative bandwidth of one-eighth octave was maintained, corresponding to a constant $Q=11.5$. As pitch and loudness gestures both concerned a single continuous sound, the beginnings and ends were tapered off by 15-ms raised-cosine amplitude envelopes to avoid discontinuities.

Table 1 displays the common signal properties for the filter’s center frequency, the presented sound pressure level (SPL) and two possible gesture durations in bold, which define those signal properties that remained constant while another auditory parameter was studied. For instance, pitch and tempo gestures were always presented at 64 dB SPL, whereas loudness and tempo gestures always concerned 1 kHz filter centre frequency. Likewise, pitch and loudness gestures were studied for two durations that matched the *slower* and *faster* tempo gestures under study.

#### Pitch

Pitch gestures comprised a continuous glide in frequency that spanned two octaves, in which frequency^2^ varied between individual audio samples along normalized time, $t = [0, 1]$. As shown in Table 1, these two-octave gestures occurred below and above the 1 kHz anchor, corresponding to *lower* and *higher* pitch ranges, respectively. In addition, pitch gestures also occurred in *ascending* and *descending* orientations and for a *shorter* and a *longer* duration, where both distinctions served as independent variables (IVs).

**Fig. 1:**
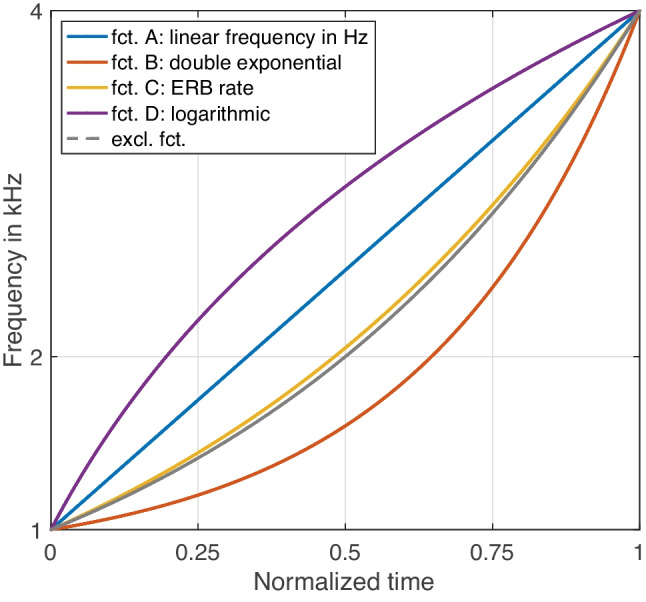
Functions A–D studied for pitch gestures. The displayed functions correspond to the *higher* frequency range and *ascending* orientation. See Appendix for an explanation concerning the “excluded function”

Four acoustic functions, labelled A to D, studied how the filter’s centre frequency, representing perceived *pitch*, varied between the specified low and high frequencies. As shown in Fig. 1, the four functions concerned either a linear increase over time in frequency in Hz (A) or a linear increase along ERB rate (C); the latter yields an exponential increase when plotted along linear frequency. The remaining two functions concerned an exponential dependency approximately twice the curvature of ERB rate (B) and a function that formed a logarithmic trajectory (D) of inverse curvature to the third. Formulaic expressions for functions A–D are given in the Appendix.

Given the known frequency dependency for loudness, the pitch gestures were expected to fluctuate in loudness if presented at the fixed reference level of 64 dB SPL. To equalize for this concurrent variation in loudness, a 64-phon contour was considered, estimated from ISO (2003). Although these contours strictly only predict equal loudness for pure tones, even for narrow-band filtered noise, the loudness dependency across frequency was assumed to behave similarly. In a pilot experiment with six participants, four versions of the same pitch gesture (ascending, ERB rate, 1–4 kHz) with varying amplitude weightings were compared. Three comprised the gain in dB of the 64-phon contour relative to 1 kHz at full, two-third, and one-third weighting, while the fourth had no weighting applied. Participants were asked to identify the version that exhibited the least variation in loudness; their responses were tied between no weighting and one-third. As a compromise, a one-sixth weighting of the full 64-phon gain was applied to all pitch gestures for the main experiment. This amounted to amplification by 1.2 dB at 250 Hz and 0.6 dB at 1.5 kHz as well as attenuation by $-$0.6 dB at 3 kHz relative to the 1 kHz reference.

#### Loudness

Loudness gestures involved continuous amplitude envelopes that spanned an equivalent sound-level difference of 18 dB across normalized time. As illustrated in Table 1, these ramps operated at *softer* and *louder* levels around the 64 dB SPL anchor.^3^ Similar to pitch, loudness gestures occurred for both *increasing* and *decreasing* orientations and for the same two durations, again serving as IVs.

**Fig. 2:**
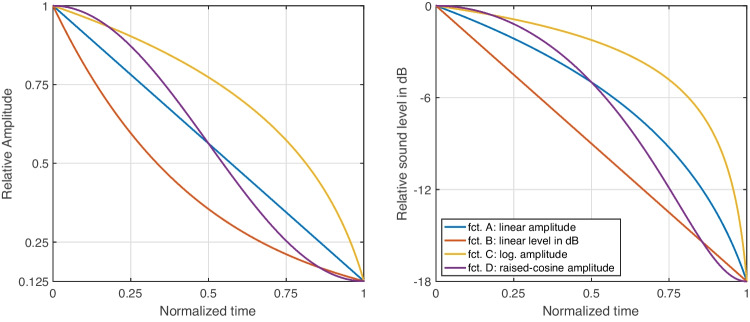
Functions A–D investigated for loudness gestures, in *decreasing* orientation. The left panel visualizes the 18-dB decrease along raw amplitude, whereas the right panel displays the same functions along relative sound level

Fig. 2 depicts the four functions that determined the change in relative raw amplitude (left panel) or equivalent change in sound level in dB (right panel) over time. The functions concerned linear trajectories along amplitude (A) and sound level in dB (B), respectively; the latter corresponds to an exponential function along raw amplitude. Function C complemented the remaining functions with an inverse, logarithmic curvature. Function D corresponded to a raised-cosine amplitude envelope or *S curve*. All four functions are common options for audio fades in digital-audio-workstation (DAW) software, e.g., *Pro Tools* (AVID, 2018), *Reaper* (Cockos Incorporated, 2018). Formulaic expressions for functions A–D are given in the Appendix.

#### Tempo

Tempo gestures were composed of a sequence of 16 noise bursts whose tempo between beginning and end changed by a factor of two, relative to the anchor tempo of 360 ‘bursts’ per minute (BPM). Each burst concerned an identical 90-ms noise segment with a Gaussian amplitude envelope. Tempo gestures could either *accelerate* or *decelerate*^4^ and occurred at two levels, *slower* and *faster*.

**Fig. 3:**
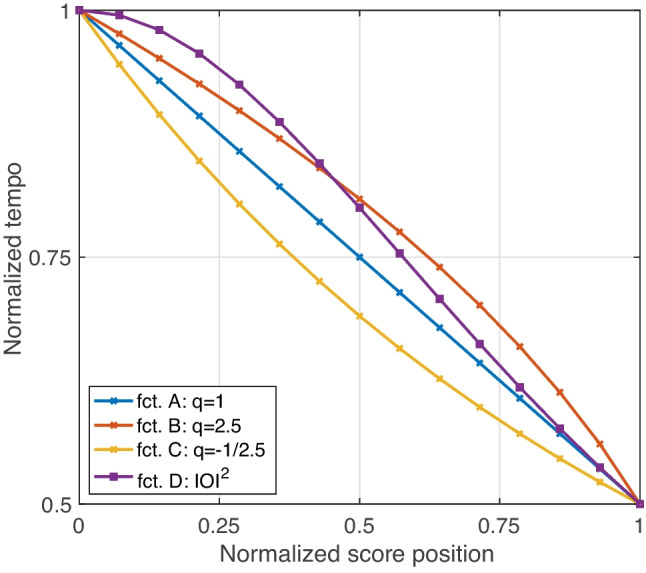
Functions A–D investigated for tempo gestures, in *decelerating* orientation from normalized full to half tempo. The 15 data points correspond to IOIs between the 16 bursts of a gesture

The functions were gathered from previous studies on tempo *ritardandi* or decelerations, expressing tempo or velocity *v* as a function of normalized score position or time. For 16 noise bursts, tempo change was modeled by the 15 inter-onset intervals (IOIs) between them, as shown in Fig. 3; IOI inversely relates to BPM. Accelerating gestures used the same functions but reversed. The first three functions employed the 1/*q* power expression proposed by Friberg and Sundberg (1999): $q=1$ (A) represented a linear function; $q=2.5$ (B) was located between the two integer values found by Friberg and Sundberg to be judged as most ‘musical’, and $q=-1/2.5$ (C) mirrored the former with an inverse curvature. Function D employed the quadratic IOI function by Repp (1992). Formulaic expressions for functions A–D are given in the Appendix.

For all functions, the first and last IOI (each involving two bursts) had to match exact values for the BPM range in question. Given these constraints and the different timing patterns for the remaining 12 bursts, the tempo gestures slightly varied in total duration. For the *slower* gestures, the total variation amounted to just under 0.4 s, whereas for *faster* gestures, the variation lay just below 0.2 s. The median duration of these tempo gestures per tempo level, namely 3.51 s and 1.8 s, served as the two durations also studied for pitch and loudness gestures, which allowed comparable durations for all gesture types.

### Experimental procedure

The experiment took place in a relatively sound-absorbent and -isolated booth (volume: 15.4 m$^3$, reverberation time: $T_{30}\!=\!0.45$ s). The booth was primarily used as a 5.1-surround sound editing and mixing suite and, apart from the loudspeakers, was equipped with two computer flat screens, mouse, and keyboard, standing on a table situated in the center of the room. The sound stimuli were presented via the center speaker of the 5.1 setup, a single Genelec *8040A* active loudspeaker. Participants faced the loudspeaker on-axis at a distance of about 1.2 m. An RME *UFX* audio interface processed the digital-to-analog conversion using the original sample rate and dynamic resolution (see Stimuli).

**Fig. 4:**
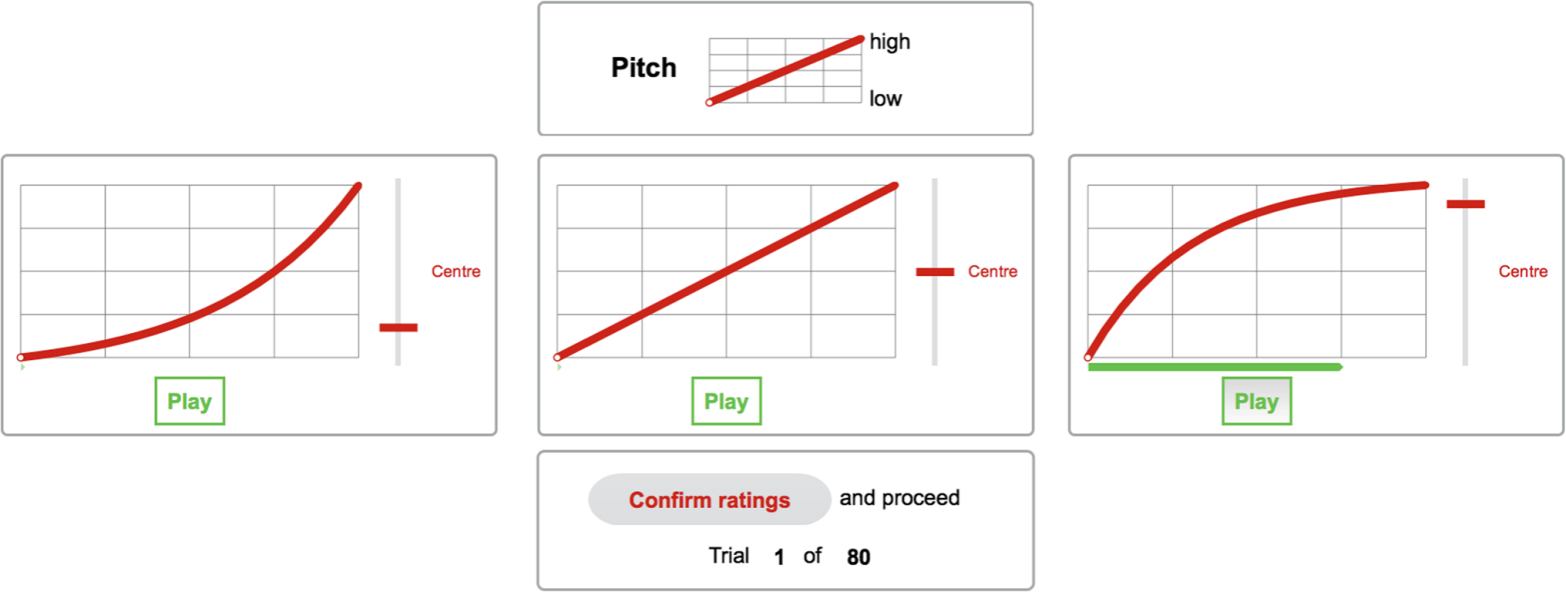
Graphical user interface (GUI) for experimental trials for the comparison of three pitch gestures (e.g., ACD). Participants could listen to each gesture in isolation and had to match the curvature of the respective auditory gesture by using the red slider. Left, middle, and right panels illustrate exponential, linear, and logarithmic curvatures, respectively

Participants were asked to match auditory gestures to an analogous visual line segment, with the x-axis reflecting time and the y-axis the parameter range under consideration, labeled *low* to *high* for pitch, *soft* to *loud* for loudness, and *slow* to *fast* for tempo. The graphical user interface (GUI) and experimental environment was implemented in Max 8 software (Cycling ’74, 2018). Using a rating slider next to the visual trajectory, as shown in Fig. 4, participants were asked to match their perception of auditory gestures to the visual analogue. Across the slider range [$-$0.7,+0.7], the line segment could assume varying degrees from exponential to logarithmic curvature; the central 0.0 value yielded a straight line. Slider positions below the center yielded downward, exponential curvature, whereas slider positions above the center yielded upward, logarithmic curvature.^5^

In a single trial, participants compared and rated a triplet of the acoustic functions A–D, i.e., occurring in the four contexts ABC, ABD, ACD, or BCD. The visual position of each function was randomly assigned, e.g., C-A-B vs. B-C-A for left-middle-right positions. Participants could listen to the individual gestures in any order and as many times as needed, with at least one listening per gesture option being required. During audio playback, a green progress bar tracked the current time position along the x-axis for better reference (see right panel in Fig. 4).

Trials for pitch, loudness, and tempo gestures were tested in separate blocks, with their order counter-balanced across participants. Trials for pitch and loudness gestures covered the IVs across 4 contexts $\times$ 2 orientations $\times$ 2 durations $\times$ 2 ranges. Tempo gestures included all IVs except for the last, because the distinction between two tempo ranges was already accounted for by the two durations. Within each block of a gesture parameter, all conditions were randomized. In total, the experiment comprised 80 trials, with a short break scheduled midway. Ahead of the main experiment, participants were familiarized with the experimental task by undergoing at least nine practice trials under the supervision of the experimenter. The nine practice trials comprised three representative examples for each gesture parameter, spanning all frequency and sound-level ranges, durations, and orientations. The median duration to complete the main experiment, including the break, was 43 min.

### Participants

Twenty participants (15 male, 5 female) with a median age of 21 years (total range: 18–51, inter-quartile range: 10.8) completed the experiment. They were recruited from the wider community of De Montfort University through cross-departmental advertisements that targeted all age groups and academic backgrounds. Two participants reported pre-existing hearing issues, both concerning tinnitus. This condition was assumed to not have critically impaired the discrimination of time-variant properties along the auditory parameters studied. Four participants classified themselves as non-musicians, whereas the remaining nine and seven participants considered themselves as amateur and professional musicians, respectively. Participation in the experiment involved informed consent, and the procedure had received prior approval by the Research Ethics Committee of De Montfort University. Participants were offered remuneration for their involvement.

### Statistical power and accuracy

Budgetary constraints imposed the limitation to 20 participants. This sample size was nonetheless expected to yield sufficient statistical power to detect anticipated effects in a repeated-measures design. Given the lack of comparable studies, as a minimum effect size, a mean slider-range difference of 0.1 between functions A–D was assumed. Across the total scale range [$-$0.7, +0.7], this yielded differences in visual curvature that were deemed as the smallest meaningful change. For power analysis, a standard error of one-third of the mean difference, that is, 0.033, was assumed, which in turn was used to estimate the population’s standard deviation. Together, this yielded estimates for statistical power of .99 and .81 for analysis of variance (one-way, four independent groups) and *t*-test (paired comparison), respectively. The 95% confidence interval (CI) around the minimum effect size was estimated as CI $= [0.01, 0.19]$.

**Fig. 5:**
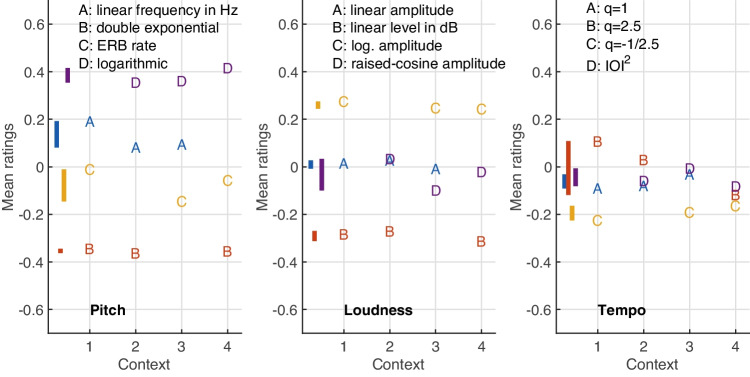
Mean shape-related ratings for the functions A–D across the triplet contexts ABC, ABD, ACD, and BCD, for pitch, loudness, and tempo gestures (left to right panels, respectively). The vertical bars on the far left of a panel illustrate the range of mean ratings for a particular acoustic function A–D across the three contexts it occurred in. See legends for key on what functions A–D correspond to for each parameter

## Results

Of main interest, pitch, loudness, and tempo gestures were each studied across four scaling variants of a relevant physical property, assumed to be the primary factor influencing gesture shape. More specifically, pitch gestures varied across frequency functions; loudness gestures varied across amplitude functions, whereas tempo gestures varied across time functions, each function denoted A to D. In order to conduct a more rigorous assessment of perceptual differences among the four functions, they were evaluated across varying triplet contexts, different ranges along their relevant physical property and/or duration, and their orientation.

Variation across triplet contexts (e.g., ABC, ACD) has to be addressed first, because the comparison of all four functions can only be conducted after averaging across contexts. Only if acoustic functions yielded perceptual differences that remained robust across contexts, their reliability across the remaining factors would be of value for further interpretation. As a result, the presentation of results focuses first on contextual variation in the next sub-section and, where robustness across contexts applies, continues with the remaining factors in the sub-section that follows.

### Contextual variation among acoustic functions A–D

For each parameter, the functions A–D were evaluated in the four triplet contexts ABC, ABD, ACD, and BCD. Fig. 5 presents the mean ratings of the four functions for pitch (left panel), loudness (middle), and tempo (right) gestures, which in turn controlled the curvature of the visual analogue. These evaluations concerned global means aggregated across the conditions listed in Table 1, both orientations, and all participants ($N=160$ for pitch and loudness, $N=80$ for tempo).

Perception of gesture shape between functions A–D that was robust to contextual variation was assumed valid when 1) the differences among A–D across the rating-scale range [$-$0.7, +0.7] were clear and encompassed at least a quarter of the scale range (exceeding three times the anticipated minimum effect size), 2) the variation across contexts was limited to a quarter of the scale range, and 3) when the functions’ ratings reflected a consistent order across contexts, for instance, by fulfilling D $\ge$ A $\ge$ C $\ge$ B (see leftmost panel in Fig. 5).

For pitch gestures (left panel in Fig. 5), all three requirements were met. First, clear differences between the functions A–D were perceived, spanning a relatively wide range ($-$0.4 to 0.4). Second, the variation of ratings across contexts was limited (see colored vertical bars), only affecting those functions assuming intermediate positions, A and C, whereas the remaining functions located at the extremes remained stable. Third, a consistent order based on the relationships D > A > C > B applied to all contexts.

Also loudness gestures (middle panel) yielded clear differences among the four functions, albeit with a narrower range ($-$0.25 to 0.25) than for pitch gestures. In addition, all functions except D exhibited little variability across contexts. Although for context ABD the mean rating for D was indistinguishable from A, $t(159)=0.81$, $p=.419$, this still yielded the consistent order C > A $\ge$ D > B across all contexts.

Tempo gestures (right panel) exhibited only weak and inconsistent differences across contexts. The perceived differences spanned less than half the range for pitch gestures (compare right to left panel). In addition, the functions A, B, and D assumed overlapping value ranges. Moreover, across contexts, inconsistent orders of functions emerged, (e.g., B > D > A vs. D > B > C). Only function C appeared to yield more reliable perceptual differences across contexts.

Overall, robust perception of gesture shape for functions A–D across contexts seemed warranted for pitch and loudness gestures but not for tempo gestures. As a result, the latter will be excluded from subsequent analysis of additional factors, as the obtained perceptual differences appear weak and inconsistent across contexts. This did not allow extrapolating reliable relationships between all four functions A–D from the triplet contexts evaluated here.

**Fig. 6:**
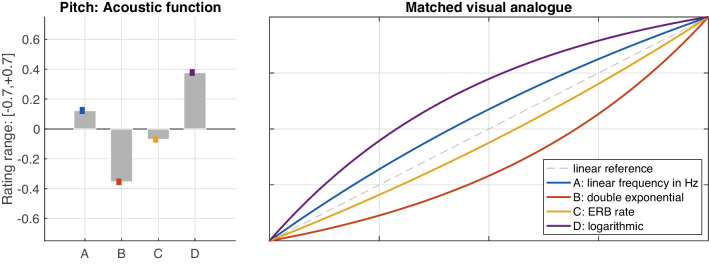
Mean ratings and standard errors for pitch-gesture shape across the four functions A–D (left panel) and the corresponding visual analogues that participants used to match the auditory shape (right panel)

### Acoustic functions A–D across different factors (IVs)

Given the robustness of pitch and loudness gestures to contextual variation, the experimental data for individual functions A–D were averaged across all three triplet contexts to yield a single value per participant, while retaining separate cases across the IVs under study here. For each parameter, repeated-measures analyses of variance (ANOVA) with the three IVs *range*, *orientation*, and *duration* were conducted. In all cases, the within-subjects residuals across all experimental conditions did not indicate departures from normality (Shapiro-Wilk test). A criterion significance level of.05 was assumed for all hypothesis tests. Where applicable, violations of sphericity (Mauchly’s test) led to adjustments of the degrees of freedom based on the Greenhouse-Geisser correction ($\varepsilon$). Effect sizes for ANOVA concern generalized eta-squared $\eta ^2_G$ (Olejnik & Algina, 2003; Bakeman, 2005).

The observed statistical power exceeded the predictions (compare to Method): The largest standard error for a main effect amounted to 0.022, yielding power estimates of $\ge .99$. The corresponding 95% CI around means can be assumed as $M\pm .05$; note that all following figures report the corresponding standard error.

#### Pitch gestures

The influence of frequency scales or functions (A–D) on the perceived shape of pitch gestures was of primary interest. Since the slider ratings, which served as the numerical dependent variable, were directly informed by the degree of curvature of the visual analogues, the presentation of results emphasizes these visual analogues to better illustrate the shape-related implications.

As shown for the mean ratings in Fig. 6 (left panel), strong differences in perceived gesture shape emerged among the frequency functions, $F(1.3,24.5)=95.00$, $\varepsilon =.43$, $p<.001$, $\eta _G^2=.52$, which suggests that functions were well distinguished perceptually. The right panel of Fig. 6 illustrates the corresponding visual analogues, which participants would have seen for the mean ratings depicted on the left. These analogues illustrate that functions resulted in varying types of perceived curvature: The logarithmic (D) and double-exponential (B) frequency scales were both matched to analogous visual shapes. Linear frequency in Hz (A) and ERB rate (C) assumed intermediate positions closer to the ideal straight line. Notably, ERB rate matched the linear trajectory more closely.

**Fig. 7:**
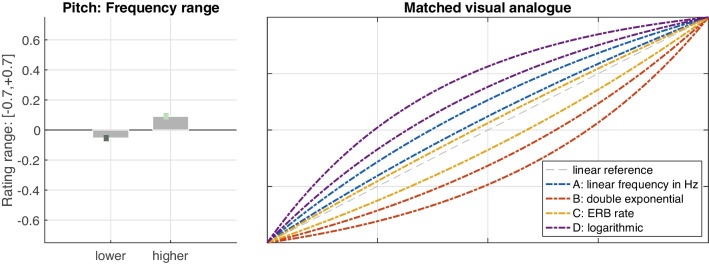
Mean ratings and standard errors for pitch-gesture shape for *low* and *high* frequency ranges (left panel) and equivalent variation applied to functions A–D (right panel). Curve pairs of identical color delimit the interval of variation; the bottom curve corresponds to the 250–1,000 Hz range, whereas the top curve relates to the 1–4 kHz range

**Fig. 8:**
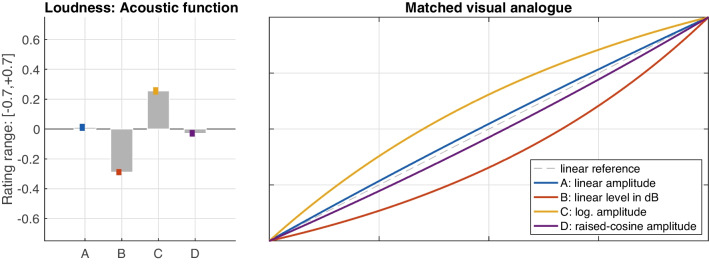
Mean ratings and standard errors for loudness-gesture shape across the four functions A–D (left panel) and the corresponding visual analogues that participants used to match the auditory shape (right panel)

Across the remaining IVs, only frequency *range* affected the perception of shape, $F(1,19)=14.00$, $p=.001$, $\eta _G^2=.08$. As shown in Fig. 7 (left panel), pitch gestures at the *higher* frequency range on aggregate yielded a slightly more positive rating than for the *lower* range, which was slightly negative. In absence of interaction effects, these differences can be assumed to apply to functions A–D to equal degree. Fig. 7’s right panel visualizes these functions across an interval that reflects the variation between both frequency ranges (bottom: 250–1,000 Hz, top: 1–4 kHz): linear frequency in Hz (A) matched the straight line more closely at the lower frequency range, whereas in the upper range, ERB rate (C) approximated the linear trajectory even more closely. Overall, ERB rate encompassed the idealized linear reference when comparing between the two frequency ranges.

**Fig. 9:**
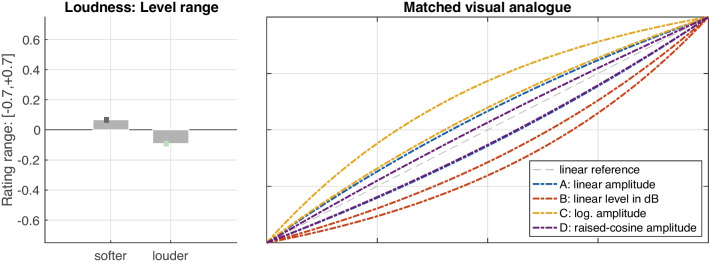
Mean ratings and standard errors for loudness-gesture shape for *soft* and *loud* sound-level ranges (left panel) and equivalent variation applied to functions A–D (right panel). Curve pairs of identical color delimit the interval of variation; the top curve corresponds to the 46–64 dB (SPL) range, whereas the bottom curve relates to the 64–82 dB (SPL) range. Differences in interval width reflect a *range* $\times$ *function* interaction

**Fig. 10:**
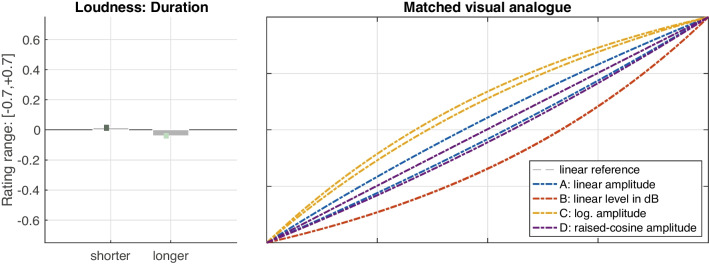
Mean ratings and standard errors for loudness-gesture shape for *short* and *long* durations (left panel) and equivalent variation applied to functions A–D (right panel). Curve pairs of identical color delimit the interval of variation; the top curve corresponds to the 1.8 s duration, whereas the bottom curve relates to the 3.5 s duration. Differences in interval width reflect a *duration* $\times$ *function* interaction

#### Loudness gestures

Also loudness gestures exhibited clear differences across the amplitude scales or functions considered, $F(1.1, 21.7)=60.80$, $\varepsilon =.38$, $p<.001$, $\eta _G^2=.37$, as illustrated by the obtained mean ratings in Fig. 8 (left panel). In terms of gesture shape (right panel), logarithmic amplitude scaling (C) was matched to an analogous visual shape, whereas conversely, a linear increase in dB level (B), which reflected an exponential increase along amplitude, was matched to an exponential curve. Linear amplitude (A) was matched most closely to an ideal straight line, although the raised-cosine function (D) approximated it as well. In sum, the shapes of functions B and C were perceived as clearly different from the shapes related to A and D. The distinction between the latter two was far less pronounced, although still reliable, $t(159)=2.03$, $p=.044$.

Unlike for pitch, the shape of the loudness gestures across A–D varied with several IVs. The first IV concerned sound-level *range*, $F(1,19)=22.50$, $p<.001$, $\eta _G^2=.09$, in that *softer* levels yielded slightly more positive ratings compared to slightly more negative ratings for *louder* levels, as shown in Fig. 9 (left panel). This difference was not equal across functions A–D, however, due to a corresponding interaction between the two factors, $F(3,57)=5.80$, $p=.002$, $\eta _G^2=.01$. Fig. 9 (right panel) illustrates the differential effect of level range on the functions A–D, again using curve pairs spanning the interval of variation: whereas amplitude in dB scaling (B) varied the least, logarithmic (C) and linear amplitude (A) functions exhibited about twice the variation, with the magnitude of variation for raised-cosine amplitude (D) falling in between.

**Fig. 11:**
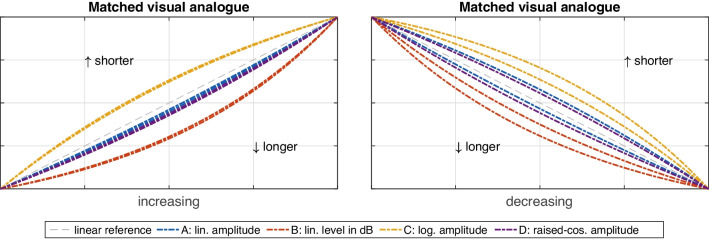
Variation of loudness-gesture shape resulting from an *orientation* $\times$ *duration* interaction applied to functions A–D. Left and right panels correspond to *increasing* vs. *decreasing* orientations. Curve pairs of identical color delimit the interval of variation along *duration*; the top curve corresponds to the *short*, 1.8 s duration, whereas the bottom curve relates to the *long*, 3.5 s duration (see arrows)

**Fig. 12:**
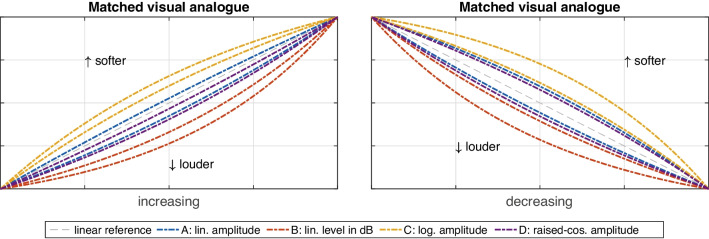
Variation of loudness-gesture shape resulting from an *orientation* $\times$ *range* interaction applied to functions A–D. Left and right panels correspond to *increasing* vs. *decreasing* orientations. Curve pairs of identical color delimit the interval of variation along *range*; the top curve corresponds to the *soft*, 48-64 dB SPL range, whereas the bottom curve relates to the *loud*, 64–82 dB SPL range (see arrows)

The second IV that affected gesture shape across functions A–D concerned *duration*. On aggregate, *longer* gestures exhibited slightly more negative ratings compared to the *shorter* gestures, $F(1,19)=6.00$, $p=.025$, $\eta _G^2=.01$, as shown in Fig. 10 (left panel). Again, an interaction with *function*, $F(3,57)=2.80$, $p=.046$, $\eta _G^2<.01$, introduced a differential effect across functions A–D: duration hardly had any effect on dB-scaled amplitude (B), whereas it introduced some variation in logarithmic (C) and raised-cosine (D) scalings of amplitude. Linear amplitude (A) exhibited the widest degree of variation; it was perceived as more logarithmically shaped for shorter gestures, whereas longer gestures yielded a more exponential curvature.

The factors *range* and *duration* also interacted with the *orientation* of loudness gestures. The *orientation* $\times$ *duration* interaction involved an asymmetry between *increasing* and *decreasing* loudness gestures, $F(1,19)=5.00$, $p=.038$, $\eta _G^2=.02$. As shown in Fig. 11, the shape of increasing gestures hardly varied between the two durations (left panel). By contrast, for decreasing gestures, the shape for all functions became relatively more exponential for *longer* gestures and conversely more logarithmic for *shorter* gestures (right panel). The second interaction reflected a similar asymmetry between *orientations*, this time with sound-level *range*, $F(1,19)=6.30$, $p=.021$, $\eta _G^2=.01$. Here, orientation affected the range of variation between *softer* and *louder* gestures, as illustrated in Fig. 12. The variation was markedly more pronounced in *decreasing* (right panel) compared to *increasing* (left panel) loudness gestures.

## Discussion

This study focused on perceptual differences in shape for uni-directional sound gestures, which concerned the auditory parameters pitch (or timbral brightness), loudness, or tempo. For each parameter, listeners compared four gesture shapes and had to describe their time course as being perceived as more linearly, logarithmically, or exponentially shaped. In other words, these shapes concerned parameter changes at constant slope or varying slopes from steep to gradual or gradual to steep.

With the aim to characterize the perception of gesture shape comprehensively, a range of potential factors of variation was considered. For one, the robustness of differences in gesture shape was evaluated as a function of contextual variation and increasing vs. decreasing time orientations to reveal possible asymmetries in their perception. Furthermore, the gestures were also assessed across wider parametric ranges, such as a total of four octaves across frequency, 36 dB across sound level, and two gesture durations. The findings for each parameter are first discussed separately before common issues are identified.

### Pitch gestures

The perception of shape in pitch gestures appeared robust, supported by strong perceived differences among the four functions A–D and consistent results across contexts. The observed moderate shifts in gesture shape across contexts for functions in intermediate locations (see A and C in Fig. 5, left panel) likely concerned a recalibration against changing frames of reference. In other words, the presence or absence of the more stable functions B and D in triplet contexts evoked relative shifts in perceived gesture shape for functions A and C.

For gesture shape averaged across contexts, the perceived differences among all four functions yielded strong effects, which suggests a highly reliable distinction between the four types of pitch gestures considered. These differences moreover remained robust to all except one of the independent variables, namely, frequency *range*. Notably, the effect of *range* was much smaller in comparison ($\eta _G^2=.08$ for *range* vs. $\eta _G^2=.52$ for *function*).

Nonetheless, the variation introduced by *range* suggests that gesture shape varied across frequency, in that all functions were perceived as relatively more exponential between 250–1,000 Hz compared to 1–4 kHz. This points toward a frequency dependence, for which a number of psychoacoustic scalings of pitch could provide explanations. The Mel scale (Stevens, Volkmann, & Newman, 1937), for instance, relies on a so-called break frequency, below which the frequency scaling of pitch is mainly linear, whereas above which the scaling becomes logarithmic. Since the lower range 250–1,000 Hz probably included this break frequency (e.g., 700 Hz in O’Shaughnessy, 1987), perceived gesture shape may have covaried with this transition in psychoacoustic scaling below and above the break frequency. Also the Bark scale (Zwicker & Fastl, 1999), used in early models of auditory filters, concerns a linear scaling in absolute 100-Hz bandwidths below 500 Hz, whereas filters above 500 Hz widen with increasing frequency.

The relevance of the Mel scale to musical applications has been contested, however, e.g., in transpositions of melodic contours (Attneave & Olson, 1971). In a similar vein, it may also become less relevant here. The Mel scale was derived from static pitches, whereas gestures involve dynamic variation of pitch. For more comparable continuous pitch glides in speech prosody, ERB rate has been found to best approximate transpositions of pitch contours (Hermes & van Gestel, 1991), notably, even for pitches with fundamental frequencies below 250 Hz, i.e., also below known Mel-scale break frequencies. Therefore, issues related to ERB rate may be more relevant.^6^

In the stimulus design, the frequency ranges were equalized along octave scaling, which given this scale’s relevance to musical applications and acoustical measurement seemed justified. Its choice was also considered as unbiased relative to the four frequency scales studied. In terms of ERB rate scaling, however, the lower and upper two-octave bandwidths were unequal and amounted to ERB-number bandwidths of 8.8 and 11.5 Cams, respectively. Thus, the 1–4 kHz range could have been perceived as being about a third wider than the lower range. Importantly, this would have mainly reflected a perceived difference in gesture *magnitude*, which in turn assumed a mediating effect on the perception of gesture *shape* across the two frequency ranges, applying to all gesture functions equally.

Although no perceptual asymmetry for increasing vs. decreasing orientations was observed, a contextualization to reports of such asymmetries seems warranted: When listeners were asked to freely imagine spatial or motion cues for rising or falling pitch contours, a clear majority associated falling pitch to downward motion, whereas the association of upward motion to rising pitch was less evident (Eitan & Granot, 2006). Furthermore, for measured velocity of hand movements, motion was again found to be more pronounced for falling than for rising pitch contours (Nymoen, Torresen, Godøy, & Jensenius, 2012). Asymmetries between pitch gestures and vertical motion have also been reported for bi-directional gestures composed of either a rising-falling or the opposite falling-rising sequence (Küssner, Tidhar, Prior, & Leech-Wilkinson, 2014); greater pitch-to-elevation correlations were found for rising-falling compared to falling-rising gestures. Assuming the same mapping asymmetry to underlie all three examples, in the third example, the recency and possible greater salience of the latter segment (rising-*falling* vs. falling-*rising*) could have mattered. Taken together, all three studies argue for more consistent mapping of decreasing pitch gestures to downward motion than vice versa.

The lack of a comparable asymmetry in the current findings may be explained by emphasizing one major difference to how the cited examples were conducted: participants in these three studies were free to express vertical motion in terms of magnitude, where the magnitude difference and/or derivative correlation measures revealed the asymmetry. In the current study, by contrast, the vertical extent from *low* to *high* was equal for both increasing and decreasing gestures, which in theory did not allow differences in gesture magnitude to matter. Therefore, since the current findings emphasized the evaluation of gesture shape and the four functions retained their relative shape regardless of increasing vs. decreasing pitch orientations, previous reports of asymmetries for pitch gestures may be limited to differences along gesture *magnitude* and may not extend to gesture *shape*.

The robust perception of pitch gestures in terms of their shape can be summarized as follows: All four functions could reliably be distinguished perceptually, even in face of the variation between 250–1,000 Hz and 1–4 kHz ranges, as illustrated in Figs. 6 and 7. Frequency functions that were based on clearly logarithmic or exponential frequency scalings assumed analogous visual shapes (compare Figs. 6 and 1). With regard to which function was perceived as the most ‘linear’, i.e., relating to a constant slope of change, ERB rate seems the closest candidate, notably, as it also encompasses the linear reference in Fig. 7. From an auditory perspective, ERB rate resembling the shape of a straight line is further supported by its approximation of the frequency scaling of human hearing (Moore & Glasberg, 1983), its superior fit in modeling pitch contours compared to purely linear or logarithmic scales (Hermes & van Gestel, 1991), and its apparent utility in visualization of speech intonation based on straight line segments (Hermes, 1998).

Conversely, a linear scaling along Hz was perceived as moderately logarithmically shaped. Despite the lack of comparable studies on gesture shape, some parallels to qualitative time-series data between pitch and vertical-motion gestures provide anecdotal support: for instance, linearly scaled rising-falling frequency trajectories corresponded to an upward, logarithmic curvature for vertical hand movement (see Fig. 2 in Küssner, Tidhar, Prior, & Leech-Wilkinson, 2014). Similarly, a non-linear transformation of vertical position, compared to no transformation, yielded a better fit to time-varying timbral brightness (see Fig. 6 in Nymoen, Caramiaux, Kozak, & Torresen, 2011). In sum, auditory linearity for pitch gestures therefore seems to require a non-linear scaling along Hz, where among non-linear options for pitch glides, ERB rate provides a better fit than a logarithmic scaling in semitones (Hermes & van Gestel, 1991).

### Loudness gestures

Also loudness gestures across the functions A–D yielded reliable perceptual differences across contexts. As for pitch gestures, the observed contextual variation likely concerned a recalibration across different frames of reference (see Fig. 5). Notably, the functions D and A were indistinguishable in one context, possibly owing to both functions exhibiting the greatest similarity and proximity along amplitude (compare to Fig. 2).

For gesture shape averaged across contexts, the perceived differences among functions A–D again reflected clear distinctions, albeit with a lower effect size than for pitch gestures ($\eta _G^2=.37$ vs. $\eta _G^2=.52$ for pitch). Unlike for pitch, however, the perception of gesture shape varied across a number of main and interaction effects involving other IVs, namely, *duration*, *range*, and *orientation*. Gesture *duration* yielded the weakest main effect observed ($\eta _G^2=.02$ for *duration* vs. $\eta _G^2=.37$ for *function*). Even more important, as illustrated by Fig. 11, the effect between *shorter* and *longer* durations appeared to only apply to decreasing loudness gestures (right panel) but not increasing ones (left panel). Durations of 3.5 s resulted in more exponential (or conversely less logarithmic) gesture shape compared to 1.8 s gestures, affecting the two intermediate functions (A and D) somewhat more than those at the extremes (B and C).

Different sound-level *ranges* also exerted an influence on perceived gesture shape, with a stronger effect ($\eta _G^2=.09$). Again, the influence of *range* on gesture shape can only be understood by also considering a differential effect between increasing vs. decreasing orientations. As illustrated in Fig. 12, for decreasing orientation (right panel), gesture shape was perceived as more exponential for SPLs spanning 64–82 dB compared to a more logarithmic shape applying to the lower 46–64 dB SPL range. This variation was pronounced and applied to all functions to about equal degree. By contrast, only about half the variation occurred in increasing loudness gestures (left panel). Notably, the variation for function A was about twice as wide as for the remaining functions.

A reason for why sound-level *range* affected gesture shape may relate to the broadband spectrum of the gesture stimuli. For loudness gestures, this concerned pink noise that was band-pass filtered at a centre frequency of 1 kHz. Compared to a sine tone at 1 kHz, for instance, the perceived loudness would have depended on most, if not all, auditory filters. Given that loudness summation operates differently within auditory filters than between them (Zwicker & Fastl, 1999) and that loudness dependence on frequency is more pronounced at lower SPLs (ISO, 2003), these complex dependencies likely became relevant here. To account for this complexity, a computational model (ISO, 2017a) that also models time-varying loudness can be consulted.^7^ Loudness here concerns a ratio scale based on the unit *Sone*.

**Fig. 13:**
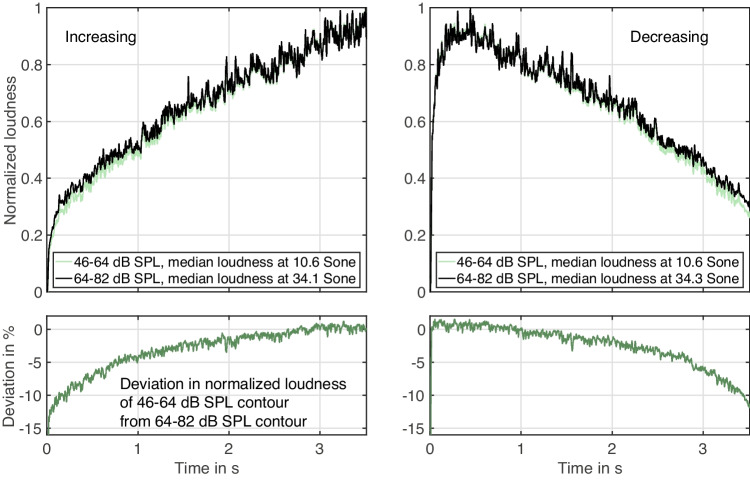
Comparison between the *soft*, 46–64 dB SPL and *loud*, 64–82 dB SPL ranges for modeled time-varying loudness, based on the standard ISO 532-1 (ISO, 2017a). The depicted loudness gestures concern linear amplitude scale (function A), 3.51-s duration, and increasing vs. decreasing orientations. Loudness in Sone was normalized to emphasize differences in gesture shape. For median absolute loudness in Sone, see legend. The bottom panels illustrate the deviation in normalized loudness of the *softer* gesture relative to the *louder* gesture

Fig. 13 shows an example of modeled time-varying loudness for gestures based on linear amplitude scaling (A) and 3.5 s duration, for both increasing and decreasing orientations. In order to elucidate differences in gesture shape, the underlying ratio scale warrants the normalization of the loudness contours. As is observable in the top panels of Fig. 13 for both time orientations, the modeled loudness exhibits a growing discrepancy between the 46–64 dB SPL and 64-82 dB SPL contours as loudness decreases. The lower panels in Fig. 13 quantify this discrepancy in relative terms, showing how the 46–64 dB SPL gestures exhibit a negative deviation of more than 10% relative to the 64-82 dB SPL gestures on the softer end, which converges toward no deviation at the louder end. In terms of gesture shape, this suggests a relatively steeper slope at the beginning for increasing gestures, or the end for decreasing gestures, which in terms of the reported findings agrees with a relatively more logarithmic shape for softer compared to louder gestures.

Although no main effect was obtained for the IV *orientation*, the difference between increasing vs. decreasing loudness gestures clearly mediated the influence of the IVs *range* and *duration*. This pattern resembles the well-established perceptual asymmetry for loudness ramps (e.g., Neuhoff, 2001; Susini, McAdams, & Smith, 2007; Ponsot, Susini, & Meunier, 2015): given equal sound-level differences for both orientations, increasing ramps are consistently perceived as louder than decreasing ramps, which applies to both sinusoidal and noise-based sounds. A recency effect for the terminating loudness in increasing ramps has been argued to bias perception on one hand (Susini, McAdams, & Smith, 2007). In the wider ecological context, on the other hand, increasing loudness can signify a potential threat, which explains its greater perceived magnitude (Neuhoff, 2001). Notably, the same asymmetry extends beyond the perception of loudness per se, as it also improves reaction times and augments neurophysiological or emotional responses (Bach, Neuhoff, Perrig, & Seifritz, 2009; Tajadura-Jiménez, Väljamäe, Asutay, & Västfjäll, 2010), which in sum points toward an enhancement across a range of perceptual abilities.

As illustrated by both Figs. 11 and 12 when comparing left with right panels, the asymmetry in the current findings manifested itself in markedly less variation along gesture durations or sound-level ranges for increasing compared to decreasing loudness gestures. In the larger context, in increasing loudness gestures (left panel), perceived differences in gesture shape among the functions A–D were clearer and more consistent, whereas in decreasing gestures, their distinction bordered on becoming blurred by the variation introduced by other factors. If increasing loudness indeed yielded greater auditory salience or acuity, as it does for factors unrelated to loudness (Bach, Neuhoff, Perrig, & Seifritz, 2009; Tajadura-Jiménez, Väljamäe, Asutay, & Västfjäll, 2010), this could also improve perceiving shape in loudness gestures. In a similar context, increasing as opposed to decreasing loudness possibly contributed to greater perceived clarity of gestural outlines conveying triangular sound shapes (Lembke, 2018). Such gesture-based spectromorphologies are common in electroacoustic music, a genre in which a greater prevalence of increasing compared to decreasing loudness contours has been observed (Dean & Bailes, 2010). In music, a preference for increasing loudness contours could therefore benefit from the increased perceptual salience and the greater expressive potential this may afford.

As the variation introduced by the IVs *range* and *duration* was narrow enough to avoid overlap of their corresponding intervals in Figs. 9 to 12, gesture shape between the functions C, B, and A/D can be assumed to have been reliably distinguished. The typical gesture shape for these three distinctions can be summarized as follows: Logarithmic amplitude scaling (C) yielded an analogous visual curvature. The exponential amplitude scaling underlying a linear change in dB sound level (B) was perceived as an analogous exponential curvature, whereas both linear (A) or raised-cosine (D) amplitude scaling approximated the straight linear reference. As these functions reflect typical control functions in waveform editors or DAWs for fade-ins or -outs, it can be argued that the visual shapes implied along raw amplitude (see Fig. 2, left panel) matches the shape of the auditory gestures. If, on the contrary, a sound engineer moved a dB-scaled level fader at constant velocity, or a straight automation line-segment was applied to the same fader, the current findings suggest that a non-linear, exponentially curved loudness contour would be perceived.

Concerning linear and raised-cosine amplitude functions, a certain perceptual ambiguity remains. A linear amplitude scaling (A) was associated to the visual linear reference consistently across contexts, and although its gesture shape appeared to vary considerably in face of other IVs, the interval of variation always centered on the linear reference. By contrast, the raised-cosine amplitude scaling (D), or S-curve, exhibited the largest contextual variation, suggesting that listeners may have experienced difficulty in expressing its shape relative to the other functions. Indeed, the remaining functions may have had the unfair advantage of exhibiting a single, consistent curvature, whereas, by design, the S-curve exhibits two opposite curvatures. Each end resembles a logarithmic or exponential curvature, while the middle portion intersects the linear function (see Fig. 2, left panel). As the S-curve would mathematically also regress onto the linear scaling, both were prone to be confused, all the more, because the visual-analogue GUI did not allow a closer fit to the S-curve. The contextual variation for D likely emerged as a consequence, especially in the longer, 3.5 s durations, in which listeners had more time to identify differences between D and A.^8^

### Tempo gestures

Unlike the two other gestural parameters, the tempo gestures studied did not yield clear and reliable perceptual differences. They were markedly weaker than differences among pitch or loudness gestures and exhibited inconsistent orderings across triplet contexts. This likens the concept of intransitivity (Tversky, 1969; Regenwetter, Dana, & Davis-Stober, 2011), where judgments for partial, pairwise contexts yield contradictions when extrapolated to the entire context. Here, intransitive relationships across triplet contexts prevented a reliable ordering among all four functions A–D, and they may have resulted from the low perceptual distinction observed. These findings suggest that no robust perception of gesture shape can be assumed from which reliable conclusions can be drawn. For instance, although function B on average best approximated ratings of 0.0, which in terms of gesture shape would relate to the linear reference, the same function exhibited the widest contextual variation across all functions.

The investigated functions stemmed from two studies that modeled tempo variations during musical performances, both in the context of *ritardandi*. Friberg and Sundberg (1999) investigated the final tempo deceleration that concludes a musical piece, whereas they did not assess whether the same models applied to equivalent tempo increases, as in *accelerandi*. Repp (1992) had previously studied the fit of parabolic, squared IOI functions on timing variations obtained from pianists, where a parabolic function modeled both the initial acceleration toward a musical phrase boundary and the deceleration that followed; thus, one function was able to model both orientations of tempo change. Furthermore, in a joint comparison of squared IOI against alternative models using the parameter *q* (Friberg & Sundberg, 1999), the use of $q =2$ and $q =3$ in the timing of musical excerpts was perceived as more ‘musical’ compared to the alternatives. Taken together, with the exception of function C, previous research suggested that the functions investigated here could be perceptually distinguished. The current findings failed to yield any reliable distinction, however, which motivates a discussion on fundamental differences, more specifically, concerning the tempo ranges and musical excerpts used.

In order to compare tempi across all studies, the traditional measure *beats* per minute will not be used, especially as it depends on the specific notated meter. Instead, *bursts* per minute (BPM) will be considered (see Method), where in the musical excerpts, *bursts* correspond to the shortest note durations (e.g., 16th notes). On this basis, tempo variations spanned decelerations from 150 to 75 BPM in Repp (1992) and 389 to 120 BPM in Friberg and Sundberg (1999), representing the highest reported tempi in both studies. In comparison, even the slower of the two tempo ranges in the current study already spanned 360 to 160 BPM for a deceleration. Half the tempi studied here were therefore considerably higher.

In addition, it will also have mattered over how many notes or bursts the tempo variations were presented. In the current study, these comprised a total of 16 bursts, which did not include a lead-in segment at constant tempo. In the two Bach preludes studied by Friberg and Sundberg (1999), by contrast, a total of five measures in either 3/4 or 4/4 meter were presented, which assumedly involved a lead-in of up to two measures. Corresponding to a typical phrase length, the subsequent two measures would have included the bulk of the deceleration and thus comprised 25 or 33 notes, respectively. For the tempo gestures studied here, the comparatively lower number of bursts and lack of a lead-in at constant tempo could have made their evaluation more difficult, hence the less reliable distinction. Notably, in Friberg and Sundberg (1999), judgments on ‘musicality’ were more pronounced for the Bach preludes than for a 21-note sequence consisting of an alternating semitone interval. Although their listeners may have judged overall ‘musicality’ as opposed to focusing how ‘musical’ only the *ritardando* sounded, it does present an example where a relatively monotonous, isochronous sound sequence yielded less perceptual distinction among tempo functions studied.

In conclusion, contrary to the findings by Friberg and Sundberg (1999), the tempo gestures between IOI square and parameters $q=1$ and $q=2.5$ were not reliably distinguished given the stimuli used here. This does not mean that reliable perceptual differences would not emerge for relatively longer and slower burst sequences. Notably, the clearest perceived differences obtained only relate to a single triplet context (see B > A > C in Fig. 5, right panel). This triplet included function C, which exhibited a contrasting, opposite curvature to the gesture for $q=2.5$, thus contributing to the only marked difference.

### Common factors

In identifying and describing differences in gesture shape across the parameters pitch, loudness, and tempo, this study aimed to cover representative ranges across frequency and sound level, while also considering time-related factors such as orientation and duration. As noted in the last section, the latter dimension was relatively short compared to the time scales for phrasing in notated, instrumental music. It should be emphasized, however, that the aim was to consider basic sound gestures that could serve as elementary building blocks, for instance, featuring as spectromorphologies (Smalley, 1997) in electroacoustic music. In the latter context, sound gestures can concern the same time scale as the motion-related actions they relate to (e.g., temporal semiotic units, Frey, Daquet, Poitrenaud, Tijus, Fremiot, Formosa, & Prod’Homme..., 2009). The utility of the functions identified therefore seem to at least apply to the timescales studied here, 1.8 and 3.5 s, whereas the unreliable findings for tempo gestures suggest that their timescales would perhaps need to be larger to convey a robust sense of gesture shape.

One overarching limitation is that all findings resulted from the comparison of triplet contexts, whereas different methods of evaluation could have been considered. For one, the perceptual evaluation of gesture shape based on a single exemplar could have been pursued. This would have assumed that gesture shape can be perceived in absolute terms; however, the observed contextual variation (see Fig. 5) suggests that gesture shape varies to some degree and may thus be better studied in relative terms. Furthermore, isolated evaluations of gesture shape were assumed to yield greater variability and likely would have required more trial repetitions to obtain reliable results. It therefore seemed more promising to rely on relative comparisons within trials. From the opposite end, an evaluation of gesture shape could also have compared all four functions jointly, but this would have prevented identifying the observed contextual variation. Duplet or pairwise contexts would have presented the remaining alternative, but considering all contexts (AB, AC, AD, BC, BD, CD) would have required twice the number of trials. Moreover, contextual shifts would have likely been more pronounced, given that duplet contexts provide a weaker frame of reference over all four functions compared to triplets. Among all options, the evaluation of triplet contexts therefore allowed the best balance between a thorough and an assumedly reliable description of gesture shape among the entire context A–D. Furthermore, although participants could evaluate a triplet context in any sequential order and repeatedly, (e.g., A-B-C-A vs. B-A-C), combined with the randomized screen location of the functions, effects of sequential order were likely specific to individuals and therefore negligible in the group averages across participants.

If crossmodal correspondences contributed to some of the observed mappings, the averaging of gesture shape across triplet contexts would only be valid if the underlying crossmodal correspondences were perceived in relative but not absolute terms. It could be argued that the observed differences in shape across frequency (for pitch) or sound-level ranges (for loudness) could have resulted from absolute and thus not transposable correspondences. Previous research, however, tends to suggest that experimental tasks based on conceptual, cross-modality matching often reflect relative modes of perception (see discussion in Walker & Walker, 2016), and even pitch-based crossmodal correspondences have been found to be perceived relatively (e.g., Brunetti, Indraccolo, Del Gatto, Spence & Santangelo, 2018). Hence, the averaging of gesture shape across triplet contexts does appear valid for the crossmodal matching task employed. Researchers on crossmodal correspondences have also stressed the distinction between metaphorical or conceptual correspondences, which can be formed voluntarily, and wholly perceptual correspondences, which arise naturally (Deroy & Spence, 2016). The latter category may explain why parameters like pitch yield stronger and more robust mappings, given their high prevalence in the real world (Parise, Knorre, & Ernst, 2014).

Another major assumption was that the chosen two-dimensional visual representation served as a reliable descriptor of the perception of gesture shape, and applied to all three auditory parameters. Concerning the assumption of the horizontal dimension to represent time, the ubiquitous use of this mapping in musical notation, graphic scores or analyses (Blackburn, 2011, 2013; Smalley, 1997), and even software user interfaces for audio production, provided strong support for its validity. Spontaneous drawings of gestural sounds have indeed shown time to be mapped to the horizontal dimension, notably, from left to right (Athanasopoulos & Moran, 2013; Engeln & Groh, 2021; Küssner & Leech-Wilkinson, 2014). With regard to the auditory parameter that best maps onto the vertical dimension (or spatial elevation), pitch served as the prime candidate (e.g., Eitan & Granot, 2006; Küssner, Tidhar, Prior, & Leech-Wilkinson, 2014; Lemaitre, Scurto, Françoise, Bevilacqua, Houix, & Susini, 2017; Nymoen,Torresen, Godøy, & Jensenius, 2012; Parise, Knorre, & Ernst, 2014; Spence, 2011; Stumpf, 1883), where the low-to-high mapping is mostly preferred, although the contrary may also be employed (Lemaitre, Scurto, Françoise, Bevilacqua, Houix, & Susini, 2017).

In terms of loudness, in two-dimensional visual reductions of musical parameters, it often finds itself sidelined by pitch or spectral frequency in assuming the vertical dimension (e.g., Blackburn, 2011; Smalley, 1997), and even in three-dimensional spectrograms, its dimension is not spatial but based on brightness or color. Research on audiovisual correspondences does also not suggest that the mapping between loudness and a single visual dimension is clear cut: whereas in the absence of variation along other auditory parameters, the vertical dimension may represent loudness (Küssner & Leech-Wilkinson, 2014), other dimensions may be preferred, like visual brightness (Marks, 1987, 1989), pen pressure in drawings (Küssner & Leech-Wilkinson, 2014) or spatial distance (Eitan & Granot, 2006; Neuhoff, 2001). Despite these diverging mapping strategies, the vertical dimension in the ubiquitous waveform display for audio signals represents amplitude or sound level and provides observers with a reliable predictor for perceived loudness variation. Moreover, this study did not aim to identify or use the most relevant audiovisual correspondence, but instead use a mapping that was readily understood. In this context, the robust findings for loudness gestures argue that this was achieved.

Unlike pitch and loudness gestures, the tempo gestures did not concern a single, continuous sound but a sequence of bursts, thus, involving multiple, discrete events. The lacking perceptual distinction among tempo gestures could therefore be linked to the visual representation of a continuous line segment being less suited. At the same time, however, the perception of tempo does not appear to rely on discrete events, as in the rate of dancers’ steps (Friberg & Sundberg, 1999) but rather on the velocity of the motion involved (Eitan & Granot, 2006; Friberg & Sundberg, 1999), where velocity more often than not corresponds to a continuous variable. It therefore remains unclear whether the visual representation as such may have also contributed to the unreliable results for this parameter.

The aim to describe the perception of sound gestures by direct association to the underlying extrasonic gestures or motions seems warranted. Hence, studies based on this assumption have been pursued and indeed succeeded in describing relevant properties of sound gestures through drawings (Athanasopoulos & Moran, 2013; Blackburn, 2013; Engeln & Groh, 2021; Godøy, Haga, & Jensenius, 2006; Küssner & Leech-Wilkinson, 2014; Merer, Aramaki, Ystad, & Kronland-Martinet, 2013), motion capture (Caramiaux, Bevilacqua, & Schnell, 2010; Küssner, Tidhar, Prior, & Leech-Wilkinson, 2014; Lemaitre, Scurto, Françoise, Bevilacqua, Houix, & Susini, 2017; Nymoen, Caramiaux, Kozak, & Torresen, 2011; Nymoen, Torresen, Godøy, & Jensenius, 2012) or self reports of mental imagery (Eitan & Granot, 2006). Compared to such free evocations along space or motion, the experimental interface in the current study was more prescriptive. By allowing only one degree of freedom, the hope was to focus the evaluation down to differences in gesture shape and curvature. One shortcoming of all the studies mentioned, including the current, however, is that auditory gesture was expressed spatially or through motion in response to its sound gesture. As perceptual asymmetries may not be ruled out, any more reliable advice or specification for visualization would require similar studies to be conducted in the reverse sense, i.e., by variably adjusting sound gestures to visualized spatial or motion cues.

Finally, if sound gestures are meaningful to both creative and functional applications, it is also important to discuss whether the degree of listener expertise and training could be of importance. Listening expertise appears to lead to clearer, more consistent mappings of spatial and/or motion cues to auditory parameters (Eitan & Granot, 2006; Küssner & Leech-Wilkinson, 2014; Küssner, Tidhar, Prior, & Leech-Wilkinson, 2014; Nymoen, Torresen, Godøy, & Jensenius, 2012). Although it was not considered as a between-subjects factor in the experimental design, the level of musical training for the participants of the current study still allows some insight. For the pitch and loudness gestures, only medium ($\rho =-.63$ ) to weak ($\rho =-.29$) rank correlations, respectively, between level of listening expertise and the intra-subject variation across triplet contexts was observed. While the potential role of listening expertise points in the right direction, it provides little explanation for the contextual variation. Lacking stronger correlations, it can be concluded that gesture shape was distinguished comparably well across a wider range of listeners.

## Conclusion

The current study shows that listeners are able to distinguish between sound gestures along the auditory parameters pitch (or timbral brightness) and loudness and to reliably express differences in perceived gesture shape based on analogous straight or curved visualizations. Given the ubiquity of graphical user interfaces for analysis or control purposes that translate between visual and auditory modalities, e.g., waveforms, spectrograms, musical notation, the current findings can inform more intuitive and accurate interfaces that are based on the underlying perceptual crossmodal mappings. Whether a graphical element is straight or curved has important implications on the expected auditory perception, from which a range of disciplines can benefit, as in the creation of music, analysis of speech prosody, and more generally a variety of sound-design applications. Ascertaining whether such mappings are in fact founded in crossmodal correspondences in the strict perceptual sense would provide the ultimate confirmation of the intuitive potential underlying these mappings, as such correspondences have been shown to provide humans with reliable bases to interact non-verbally (Schmitz, Knoblich, Deroy, & Vesper, 2021).

Alongside differences in gesture magnitude and orientation, only the consideration of shape can be argued to complete a sound gesture’s identity. Whereas orientation helps determine *what* the gesture conveys, e.g., a rise vs. fall, only by evaluating *how* the auditory parameter in question varies over time conveys a gesture’s full expressive, if not even semantic, potential. The fundamental insight gained from the uni-directional pitch and loudness gestures studied here also points toward possible interactions in the formation of gesture identity, as in possible mediating effects between gesture magnitude and shape as well as potential perceptual asymmetries between orientations.

Future studies that consider all constituent properties of sound gestures are needed to elucidate the exact nature of their interrelationship, and this could likewise shed more light on bi-directional (e.g., Küssner, Tidhar, Prior, & Leech-Wilkinson, 2014) or multi-directional gestures. This more comprehensive understanding could in turn also inform more controlled studies on when gestures may arise out of multiple concurrent, possibly incongruent auditory parameters, where exploratory studies suggest that auditory salience may play a crucial role, e.g., pitch dominating over timbral brightness (Nymoen, Torresen, Godøy, & Jensenius, 2012).

The current experimental findings illustrate how higher- or *meso*-level gesture identity depends on features encoded in the *micro*-level sonic information (Godøy, 2006). This paves the way for future investigations into *macro*-scale sonic contexts, where uni-directional gestures like those studied here would serve as basic units in longer gesture sequences or also simultaneously occurring gestures. Such gestural interplay has been argued to yield large-scale sound shapes (Blackburn, 2011; Smalley, 1997), the perceptual investigation of which would be best situated within auditory scene analysis (Bregman, 1990). While notions of gesture and shape are common to areas such as speech (e.g., Hermes, 1998) and traditional music (e.g.,Gritten & King, 2006; Leech-Wilkinson & Prior, 2017), a better understanding of their perceptual underpinnings will be of special value to modes of sonic communication that rely on less established and consistent syntactical frameworks, such as electroacoustic music or the design of auditory displays or signalization.

Concerning psychological research, the consideration of dynamic stimuli and their underlying mapping functions provides an additional perspective on auditory perception and its crossmodal correspondences. The observed relevance of a mapping function’s shape motivates its potential role to be investigated at the perceptual level using suitable experimental paradigms. Although crossmodal interference effects for congruent or incongruent mappings have been established (Deroy & Spence, 2016; Spence, 2011), the common limitation to two stimulus conditions confirmed only the effect of orientation (e.g., upward vs. downward). Based on the introduced notion of sound gestures conveying information regarding orientation but also magnitude and shape, only the additional consideration of the latter two would clarify how stimulus range and scaling affect crossmodal perception (Anikin & Johansson, 2019; Parise, 2016). Given the critical importance of time in speeded-response paradigms, a revised mode of stimulus presentation may become necessary, because whereas the evaluation of auditory shape occupies time, evaluating visual shape can occur instantaneously. This will require special attention in determining crossmodal equivalences across space and time (see Handel, 1988; Kubovy, 1988) that reflect those studied here. Expanding the previous experimental paradigm to account for the variables orientation, magnitude, and shape would shed light on how ‘straight’ or ‘curved’ sounds may contribute to crossmodal perception.

## Open practices statement

The data for the experiment are available through a Figshare repository; see URL in a section that follows. The experiment was not preregistered.

## Data Availability

The behavioural datasets generated and analysed for the current study are available in a Figshare repository, https://doi.org/10.25411/aru.c.6645653.v1.

## Appendix A: Formulas for acoustic functions

Formulaic expressions for the functions A–D for each auditory parameter are given below. All consider a continuous variable for normalized time, $t=[0, 1]$, as input.

### Pitch gestures

Pitch relates to the filter’s centre frequency *f* in Hz for the range $f_{min}$ to $f_{max}$. Eqs. 1 to 4 concern linear frequency in Hz (A), ERB rate (C), double exponential (B), and logarithmic (D) scales, respectively, ascending in time.^9^

1 $$\begin{aligned} f(t) = (f_{max}-f_{min})\cdot t+f_{min} \end{aligned}$$

2 $$\begin{aligned} f(t) =\left( 10^\frac{\left( E(f_{max})-E(f_{min})\right) \cdot t+E(f_{min})}{21.4}-1\right) \cdot \frac{1000}{4.37} \end{aligned}$$

with $E(f)=21.4\cdot log_{10}\left( 4.37\cdot \frac{f}{1000} +1 \right)$ representing ERB rate (Glasberg & Moore, 1990).

3 $$\begin{aligned} f(t) =(f_{max}-f_{min})\cdot \frac{R ^{2t}-1}{R^2-1}+f_{min} \end{aligned}$$

with $R=f_{max}/f_{min}$ representing a frequency ratio.

4 $$\begin{aligned} f(t) =(f_{max}-f_{min})\cdot \frac{log_{10}\left( (R-1)\cdot t+1\right) }{log_{10}\left( R\right) }+f_{min} \end{aligned}$$

with $R=2\cdot log_2\left( f_{max}/f_{min}\right)$ representing an octave-related frequency ratio.

### Loudness gestures

Loudness relates to the relative signal amplitude *a*, which here varies by 18 dB. Eqs. 5 to 8 concern scales of linear amplitude (A), exponential amplitude or linear sound level in dB (B), logarithmic amplitude (C), raised-cosine or S-curve amplitude (D), respectively, descending in time.

5 $$\begin{aligned} a(t) = (1-10^{-18/20})\cdot (1-t) +10^{-18/20} \end{aligned}$$

6 $$\begin{aligned} a(t) = 10^{-18/20\cdot (1-t)} \end{aligned}$$

7 $$\begin{aligned} a(t) = (1-10^{-18/20})\cdot log_{10}\left( 9\cdot t+1\right) +10^{-18/20} \end{aligned}$$

8 $$\begin{aligned} a(t) = (1-10^{-18/20})\cdot cos\left( \pi /2\cdot (1-t)\right) ^2+10^{-18/20} \end{aligned}$$

In terms of equivalent sound level in dB, the time-varying amplitude *a*(*t*) features in the expression $L_{a}(t) =20\cdot log_{10}\left( a(t)\right) +\Delta L_{p}$, with $\Delta L_{p}$ representing the necessary level gain to calibrate the presentation to the absolute SPL specified in Table 1.

### Tempo gestures

Tempo relates to normalized velocity *v*(*t*), in which the variable *t* does not represent physical time but models equally spaced note positions in a musical score. The functions below end at half the starting velocity, i.e., $v(1)=0.5$. Eq. 9 concerns the 1/*q* power expression proposed by Friberg and Sundberg (1999).

9 $$\begin{aligned} v(t) = (1+(0.5^q-1)\cdot t)^{1/q} \end{aligned}$$

The investigated exponents comprised $q=1$ (A), a linear function, $q=2.5$ (B), a positive power function, and $q=-1/2.5$ (C), the inverse of the preceding function.

Eq. 10 concerns the quadratic IOI function (D) by Repp (1992), in its *v*(*t*) derivation by Friberg and Sundberg (1999).

10 $$\begin{aligned} v(t) = \frac{1}{t^2+1} \end{aligned}$$

## Footnotes

1 Translated from the French term *unités sémiotiques temporelles*

2 Frequency is scaled in either the unit Hertz (Hz, cycles per second) or equivalent rectangular bandwidth (ERB) rate (Moore & Glasberg, 1983).

3 It should be noted that the noise floor in the sound booth yielded 44 dB SPL. However, this was almost exclusively due to the three lowest third-octave bands, centered on 20, 25, and 31.5 Hz, and should thus not have impaired the audibility of *softer* loudness gestures at their 46-dB-SPL minimum. The three bands in question exhibited levels of 41, 39, and 36 dB SPL, respectively, and the octave-band total alone amounted to 43.9 dB SPL. All higher frequency bands remained below 30 dB SPL, for which the calibrated SPL meter did not display numerical values. The SPL meter had no frequency weighting applied and used a 1-s (*slow*) averaging window.

4 As the noise burst’s signal content was not time-symmetric, both the *accelerating* and *decelerating* gestures used the same time orientation for the burst, to ensure the burst always sounded the same.

5 Max 8’s function object, which used a single line segment, served for both the GUI and data acquisition. For analysis and visualization purposes, the underlying functional dependency between the function object’s value range and the line segment’s curvature was thereupon numerically approximated in MATLAB (Mathworks, 2020). Each curvature function was sampled over 51 equi-distant steps spanning the normalized time interval [0,1]. The GUI’s rating slider spanned a range of [$-$0.7, +0.7] and allowed for a minimum resolution of 0.011, which altogether resulted in a total of 70 different curvature functions. For each of these functions, the linear correlation between Max and MATLAB implementations was close to perfect, $r(51)>.99$, $p<.001$.

6 Also recent models for auditory filters use a scaling based on ERB rate (e.g., Irino & Patterson, 2006)

7 Although Part 2 to the same ISO 532 standard (ISO, 2017b) concerns an alternative loudness model that scales auditory filters along ERB rate (Moore & Glasberg, 1983), it did not offer a standardized method to model time-varying loudness. The only available standardized method models auditory filters along the Bark scale.

8 Although not considered in the main analysis reported, a repeated-measures ANOVA was also conducted on the standard deviations for loudness-gesture ratings across triplet contexts. Function D exhibited a markedly higher standard deviation than all remaining functions, $F(3,57)=4.60$, $p=.006$, $\eta _G^2=.02$, notably, limited to the longer duration, $F(3,57)=5.50$, $p=.002$, $\eta _G^2=.02$.

## Code Availability

Analysis code in R and MATLAB languages can be made available upon request.
